# An accurate and efficient method for occlusal tooth wear assessment using 3D digital dental models

**DOI:** 10.1038/s41598-020-66534-4

**Published:** 2020-06-22

**Authors:** Nikolaos Gkantidis, Konstantinos Dritsas, Yijin Ren, Demetrios Halazonetis, Christos Katsaros

**Affiliations:** 1 Department of Orthodontics and Dentofacial Orthopedics, University of Bern, CH-3010 Bern, Switzerland; 2 Department of Orthodontics, W.J. Kolff Institute, University Medical Center Groningen, University of Groningen, 9700RB Groningen, The Netherlands; 3 Department of Orthodontics, School of Dentistry, National and Kapodistrian University of Athens, GR-11527 Athens, Greece

**Keywords:** Oral anatomy, Dental diseases, Structural materials

## Abstract

Tooth or material wear in a dentition is a common finding that requires timely diagnosis for management and prevention of further loss or associated esthetic or functional impairment. Various qualitative and quantitative methods have been suggested to measure tooth or material wear, but they present with limitations, such as imprecision, subjectivity, or high complexity. Here we developed and assessed an efficient 3D superimposition method to accurately measure occlusal tooth wear on 3D digital dental models. For this purpose, teeth on plaster casts were manually grinded on their occlusal surfaces to simulate various degrees of tooth wear. The casts were scanned using a surface scanner. Grinded tooth crowns (T1) were segmented and compared to the original crowns (T0) using five 3D surface superimposition techniques and a gold standard technique (GS). GS measurements were obtained by using intact adjacent structures as superimposition references. The technique of choice (complete crown with 30% estimated overlap of meshes) showed the best reproducibility (maximum difference < 0.050 mm^3^) and excellent agreement with the GS technique (median difference: 0.032 mm^3^). The suggested 3D superimposition method offers a highly efficient and accurate tool for tooth wear assessment, which could be applicable to clinical conditions.

## Introduction

Tooth wear in a dentition can be a result of attrition, erosion or abrasion and is expressed as loss of dental matter, especially of enamel. Timely diagnosis is necessary to manage this condition and avoid further tooth loss or other associated esthetic or functional impairment in the future^1,2^. Wear of dental materials is also critical since restorations have to be resistant to structural and form changes, without causing damage to the opposing teeth^3,4^.

Over the past few years, various qualitative and quantitative methods have been suggested to measure tooth wear. Conventional approaches, such as those of Eccles^5^, Smith and Knight^6^, the New Tooth Wear Index (NTWI)^7^, or the Basic Erosive Wear Examination (BEWE)^8^ assess dental material loss qualitatively through the use of indices. The main shortcoming of such indices is that they are subjective and their sensitivity is unsatisfactory, especially when tooth wear is assessed in relatively short time-spans^9^.

For this reason, quantitative methods on 3-dimensional (3D) dental models have been developed^10^. These are considered advantageous, since they are more objective and they provide more accurate estimation of the outcome, usually as tooth height or volume loss^11^. However, the utilization of such methods *in vivo* or *ex vivo* is usually rather complicated and time-consuming^12^, and thus not feasible in a regular clinical basis or even for research purposes, if the required expertise and special equipment are not available. To our knowledge, when considering these specialized techniques, there is only one study that used a validated technique to measure the 3D occlusal enamel wear in a clinical setting^11,13^.

The steadily increasing usage of intraoral scanners in contemporary clinical dentistry^14^ could provide the required surface models for quantitative 3D wear assessment and the available software in the market could facilitate the *ex vivo* implementation of the relevant techniques on a regular clinical setting. Previous *ex vivo* wear assessment methods relied mostly on superimposition of tooth areas that were not considered to be affected by tooth wear between two or more time points^11,15–17^. However, the selection of such areas and the validation of these methods compared to a gold standard measurement, which would provide the true value, has not been adequately investigated^11^. Numerous factors can influence wear assessment through these techniques, such as movement of teeth between two time points, the accuracy of the obtained 3D surface model, especially in areas that are inherently difficult to scan, the reference areas selected to register the serial surface models and the software settings used for the superimposition.

Thus, the aim of the present study was the development and *in vitro* assessment of the precision and trueness of a 3D superimposition method that can be used in a clinical setting to visualize and measure occlusal tooth wear in all dimensions of space.

## Materials and Methods

### Ethical approval

The research project is registered and approved by the Swiss Ethical Committee of the Canton of Bern (Protocol No. 2019-00326). The methods were carried out in accordance with the relevant guidelines and regulations. All participants signed an informed consent prior to the use of their data in the study.

### Sample

For the needs of the study, sixteen dental plaster models (type IV plaster, white colour, Fujirock EP Premium, GC, Leuven, Belgium) with (n = 8; 4 maxillary and 4 mandibular) and without (n = 8; 4 maxillary and 4 mandibular) aligned dental arches were selected from the archive of the Department of Orthodontics and Dentofacial Orthopedics, University of Bern, Switzerland. The models depicted a full permanent dentition of all natural teeth, except for third molars, and no extreme morphology of the intraoral anatomical structures, assessed through visual inspection. Models with aligned dental arches were considered those with crowding less than 1 mm, whereas the models without well aligned dental arches had crowding between 4 and 10 mm. The detailed sample composition is provided in Supplementary Table S1.

### Tooth wear simulation

Based on a predefined study setting, eighteen teeth of each tooth type (incisors, canines, premolars, and molars) that were equally distributed among the dental models (maxillary and mandibular, with and without crowding) were selected to be manually grinded on their occlusal surfaces. Various degrees of tooth wear were simulated (mild, moderate and severe, corresponding to approximately 0.5, 1, and 2 mm of vertical loss, respectively), equally distributed within each tooth type (Supplementary Table S1). The predefined amount of tooth wear was first marked on the stone casts with a black pencil and then removed using a laboratory straight handpiece or a laboratory stone knife, to simulate normal tooth wear. Teeth were grinded both symmetrically and asymmetrically, aiming to simulate a variety of actual clinical conditions (Supplementary Figure S1). This setting allowed for presence of two intact teeth adjacent to each grinded tooth. The intact teeth, along with the adjacent gingival or palatal structures that were not artificially altered, comprised the stable superimposition reference areas that provided the gold standard measurement (true value).

### 3D model acquisition

The dental casts were scanned before (T0) and after the occlusal tooth wear simulation process (T1) using a high accuracy laboratory 3D surface scanner (stripe light/LED illumination; accuracy <20 μm; Laboratory scanner D104a, Cendres+Métaux SA, Rue de Boujean 122, CH-2501 Biel/Bienne) to obtain the 3D Standard Tessellation Language (STL) models used in the study. The digitized 3D models were thereafter imported and analysed with Viewbox 4 software (version 4.1.0.1 BETA, dHAL Software, Kifissia, Greece, http://www.dhal.com/viewboxindex.htm).

### Tooth wear volume measurement workflow

The crowns of the grinded teeth (T1) were manually segmented and compared to the original crowns (T0) using five test techniques and the gold standard technique.

**Figure 1:**
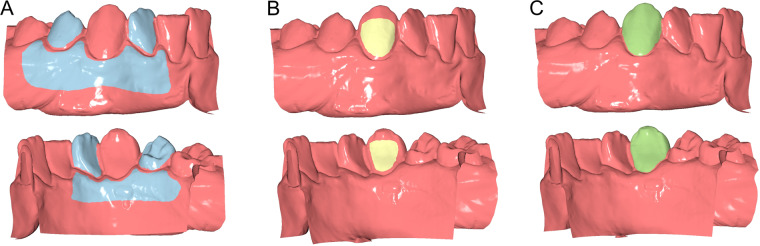
Reference areas used to measure tooth wear at the lower right canine. (**A**) Gold standard area (GS, blue). (**B**) Partial crown area (PC, yellow). (**C**) Complete crown area (CC, green). The upper row shows the buccal and the lower row the lingual view. All images were generated using Viewbox 4 software (version 4.1.0.1 BETA, http://www.dhal.com/viewboxindex.htm).

The gold standard measurements (GS) were obtained by using the adjacent intact teeth and alveolar processes as superimposition reference areas (Fig. 1A). These were unaltered, identical structures, and thus, perfect congruence of the two surface models is expected in these areas following a best-fit superimposition. Thus, the amount of occlusal wear on the tooth of interest was accurately measured as described below.

The first group of measurements (PC: partial crown) were obtained using the part of the T0 clinical crown that was considered intact as superimposition reference (Fig. 1B). For the second measurement group (CC: complete crown), the complete T0 clinical crown was used as superimposition reference (Fig. 1C).

The T0/T1 3D models of each patient were superimposed, in each reference area described above, using the software’s implementation of the iterative closest point algorithm (ICP)^18^, with different settings. In all cases, prior to the application of the ICP algorithm, the two objects were manually approximated to facilitate the automatic registration. For each registration, the whole process was repeated starting from the original initial position of the two models to be registered.

At first, the performance of three different ICP settings was tested. Setting (A) comprised: 100% estimated overlap of meshes, matching point to plane, exact nearest neighbor search, 100% point sampling, 50 iterations. Setting (B) was the same as (A), but with 80% estimated overlap of meshes. Setting (C) was the same as (A), but with the estimated overlap of meshes freely defined by the operator for each individual measurement, based on visual inspection of the overlap of the superimposed teeth, the adjacent intact structures and the relevant colour maps. The selected value for each case, obtained after various pilot superimpositions, was noted in an Excel sheet. A number close to the average of these values (average: 40.0, SD: 12.2, range: 20–75 estimated overlap of meshes) was used for a fourth setting (D), which was same as (A), but with 30% estimated overlap of meshes. We decided to use a value slightly lower than the average reported above, because further assessment of individual cases showed that the lower values worked satisfactorily in all cases and were closer to the gold standard as compared to higher values. The specific techniques tested in this study (combinations of ICP settings and reference areas) are listed in Table 1.

**Table 1:** Superimposition techniques tested in the study.

Technique	Reference area	Estimated overlap
GS	Adjacent intact teeth and alveolar processes	100%
PC(A)	Buccolingual surfaces	100%
PC(C)	Buccolingual surfaces	User defined
CC(B)	Complete crown	80%
CC(C)	Complete crown	User defined
CC(D)	Complete crown	30%

**Figure 2:**
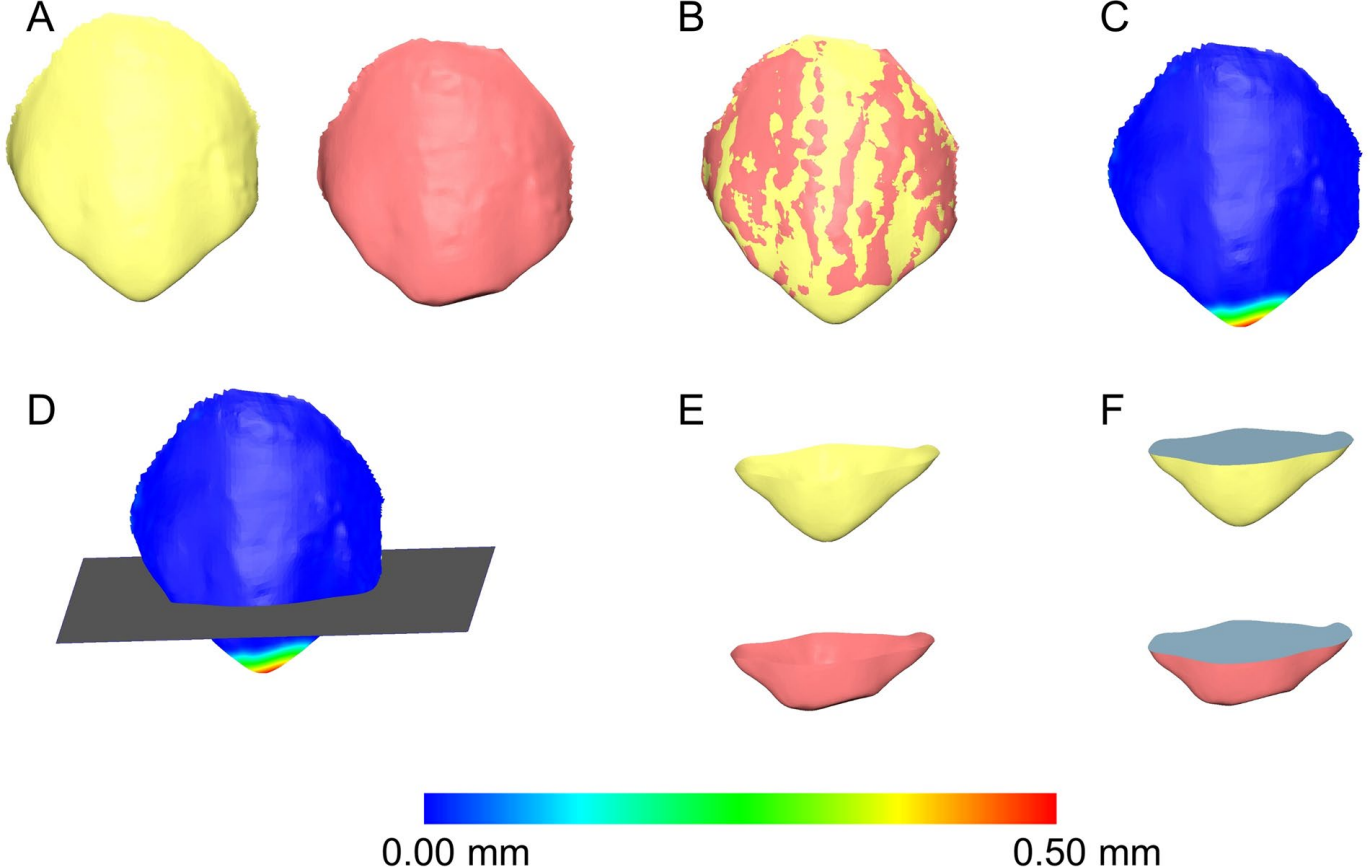
Tooth wear measurement process in an upper canine. (**A**) Tooth before (yellow) and after (red) tooth wear. (**B**) Superimposed tooth crowns using the complete crown technique and setting D. (**C**) Color coded distance map showing the tooth wear. (**D**) Level (grey) used to simultaneously slice the two crowns. (**E**) Sliced tooth crowns. (**F**) Holes filled to create watertight models, and thus, calculate volumes. All images were generated using Viewbox 4 software (version 4.1.0.1 BETA, http://www.dhal.com/viewboxindex.htm).

**Figure 3:**
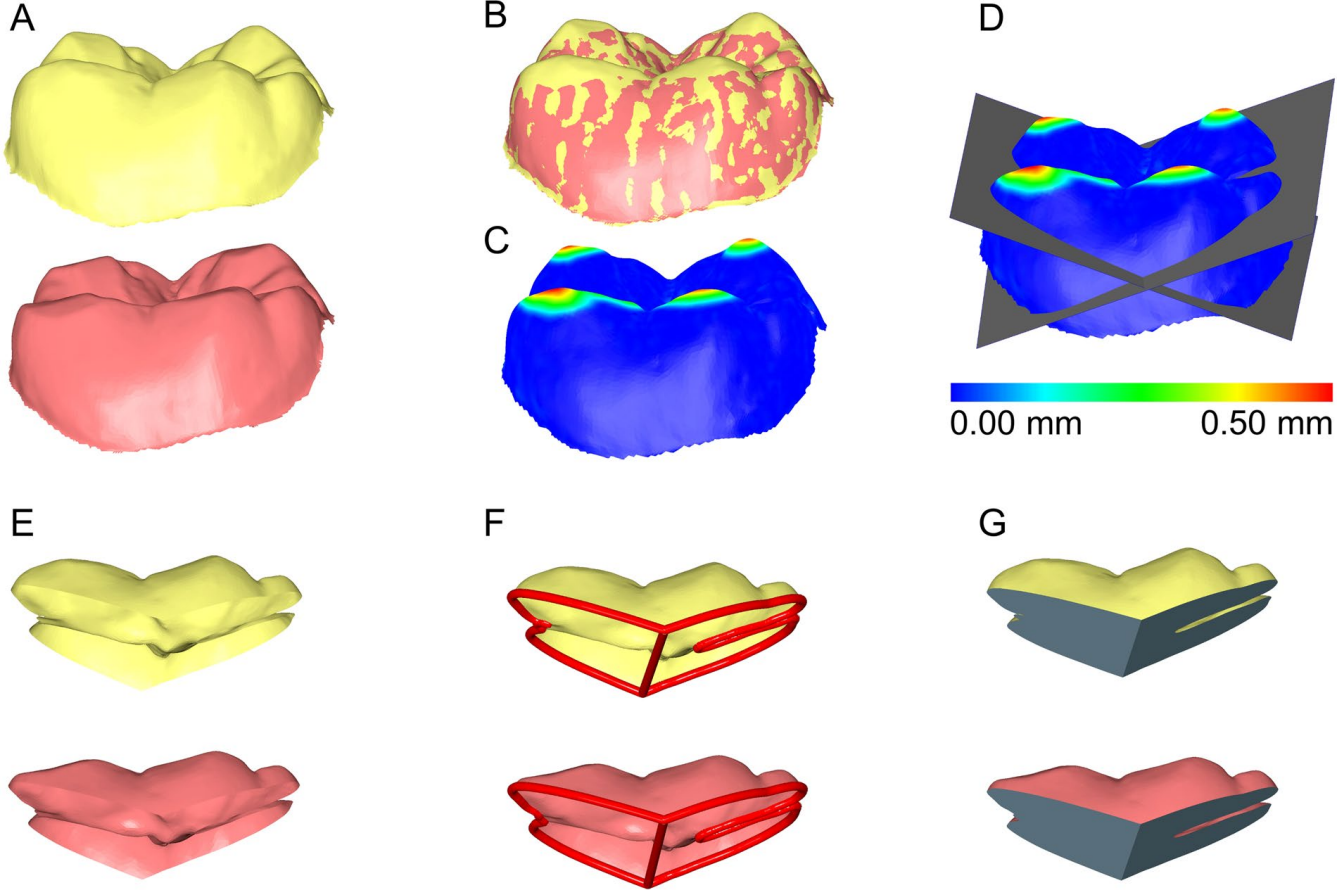
Tooth wear measurement process in a lower molar. (**A**) Tooth before (yellow) and after (red) tooth wear. (**B**) Superimposed tooth crowns using the complete crown technique and setting D. (**C**) Color coded distance map showing the tooth wear. (**D**) Levels (grey) used to simultaneously slice the two crowns. (**E**) Sliced tooth crowns. (**F**) Connected contralateral points in sharp edges (red line), splitting the hole in two parts to ensure identical hole filling process. (**G**) Holes filled to create watertight models, and thus, calculate volumes. All images were generated using Viewbox 4 software (version 4.1.0.1 BETA, http://www.dhal.com/viewboxindex.htm).

Following each superimposition, the registered 3D models of the crowns of interest were simultaneously sliced using one (gingival) to three planes (gingival, mesial, and distal), depending on the case. Prior to slicing, a colour coded distance map was created to ensure that no tooth wear surface was eliminated from the occlusal part of the tooth. The holes of each occlusal tooth part were then filled in through a specific process that ensured identical filling of both T0 and T1 tooth models (Fig. 2). To ensure that the holes got filled in exactly the same way, the contralateral points in sharp edges were connected, splitting the hole on the underside of the crowns in two or more parts. As a result, the edges of each subsequent hole lied on the same plane, so that the holes could be closed in only one way (irrespective of the software’s algorithm) to create watertight T0 and T1 3D models of each tooth that differed only in their occlusal part, but were identical otherwise (Fig. 3). Thus, the difference of the volumes of the two resulting models represented the amount of the tooth wear at the entire occlusal/incisal surface.

The amount of tooth wear that was detected through the gold standard technique and expressed as volume loss of tooth structure (mm^3^) was then compared to the test techniques.

For intra- and inter-operator error testing (reproducibility), the whole process was repeated for 20 teeth on four randomly selected models, two maxillary and two mandibular (one with and one without crowding, each) by two operators, following a 1-month washout period.

### Statistical analysis

Statistical analysis was carried out by using the IBM SPSS statistics for Windows (Version 25.0. Armonk, NY: IBM Corp). Raw data were tested for normality through the Kolmogorov-Smirnov and Shapiro-Wilk tests and did not have a normal distribution in certain cases. Thus, non-parametric statistics were applied.

Agreement between different techniques with the gold standard technique (trueness) in tooth wear assessment was shown in box plots. Zero median value implies perfect trueness, whereas the larger the deviation from zero the lower the trueness of the technique. The range of deviation of individual values from the median value within techniques shows the precision of each technique. Differences in trueness and precision among different techniques were tested in a paired manner through Friedman’s test. In case of significant results, pairwise comparisons were performed through Wilcoxon’s signed rank test.

Potential effects of presence of crowding, tooth type, or tooth wear amount on the trueness and precision of each technique were explored through visual inspection of relevant plots and unpaired mean comparison tests within techniques.

Intra- and inter-operator error (reproducibility) of each technique on tooth wear measurement was assessed through Bland Altman plots, with markers set by tooth category. Any deviation from zero shows imprecision of the technique. Differences in the reproducibility of the techniques were tested in an unpaired manner through Kruskal-Wallis test. In case of significant results, pairwise comparisons were performed through Mann-Whitney U test.

In all cases, a two-sided significance test was carried out at an alpha level of 0.05. In case of multiple comparisons, a Bonferroni adjustment was applied to the level of significance to avoid false positive results.

## Results

The hole filling process used to create watertight models was repeated in 20 randomly selected teeth and always provided identical volumes.

There was a consistent pattern of tooth wear amounts in each tooth type, measured through the gold standard technique, verifying the study setting (Supplementary Figure S2).

**Figure 4:**
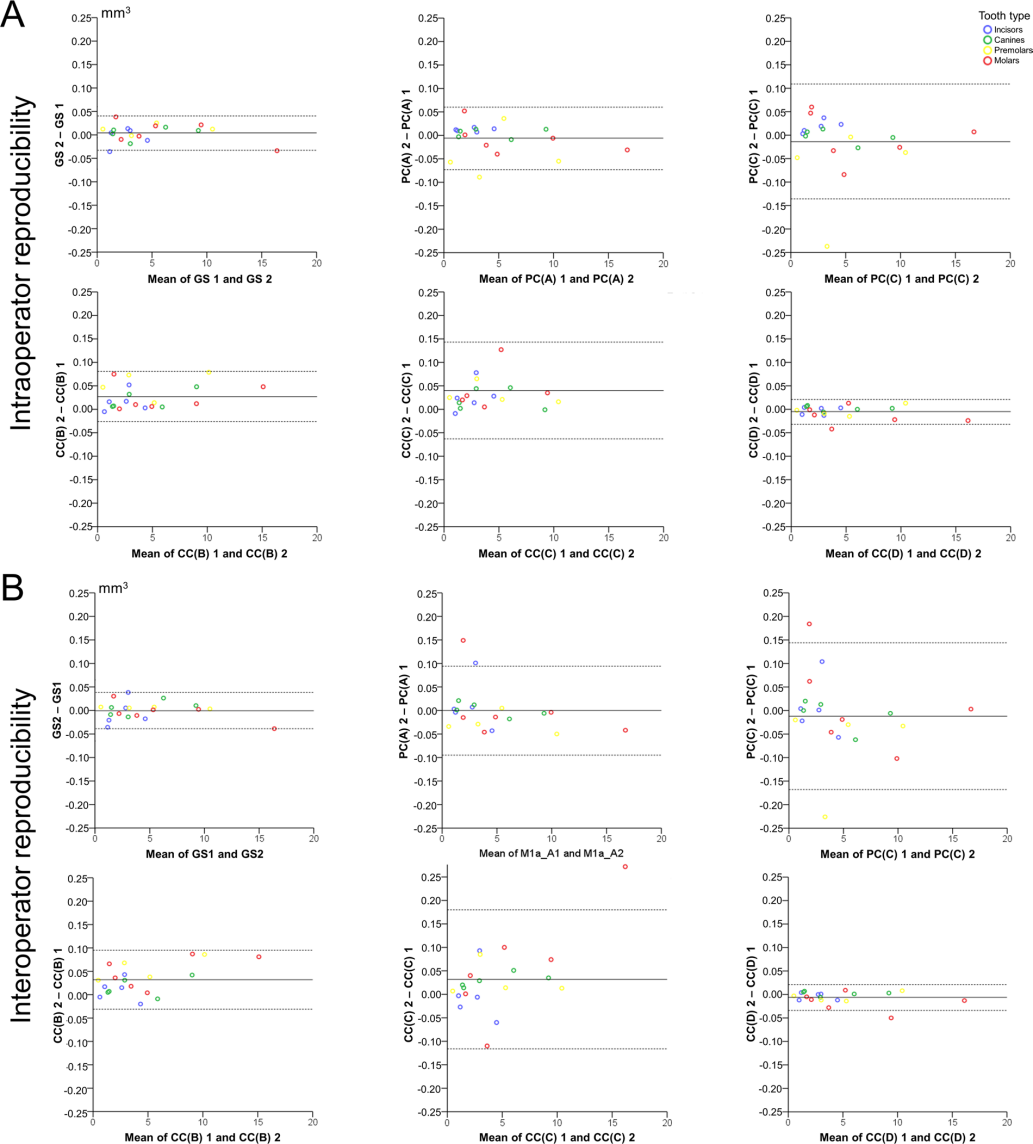
Reproducibility of tooth wear measurement with all techniques. (**A**) Bland Altman plots showing intra-operator error. (**B**) Bland Altman plots showing inter-operator error. The axes length represents the true range of measured tooth wear values. The continuous horizontal line shows the mean and the dashed lines the 95% confidence intervals.

Bland Altman plots for intra- and inter-operator error showed consistent results. There was no systematic error in any technique since mean measurements were always not significantly different from 0 (one sample t-test, p > 0.05). There was no evidence that the difference between repeated measurements was increasing by an increase in the amount of tooth wear. Tooth type also did not seem to affect reproducibility. The gold standard measurement and technique CC(D) showed the best reproducibility, overall and in individual measurements (max difference <0.050 mm^3^). Interestingly the repeatability of the CC(D) technique was slightly better even compared to the GS technique (Fig. 4). However, the GS technique showed also optimal trueness (Supplementary Figure S3).

**Figure 5:**
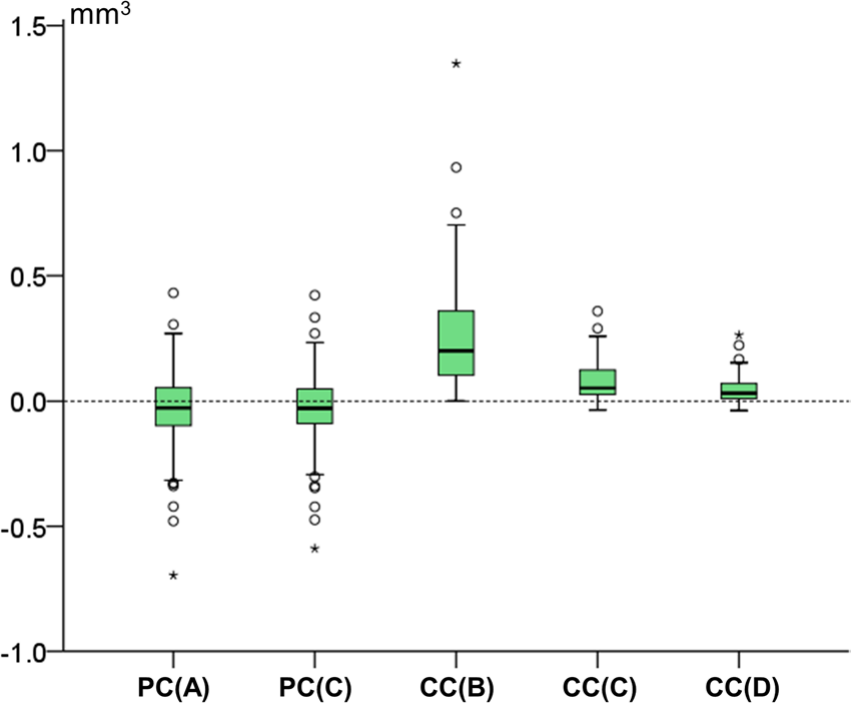
Box plots showing in the y-axis the difference of each technique with the gold standard technique in tooth wear measurements. The upper limit of the black line represents the maximum value, the lower limit the minimum value, the box the interquartile range, and the horizontal black line the median value (trueness). Zero value indicates perfect agreement with the gold standard. The vertical length of each plot indicates precision.

All tested techniques, except for PC(A) and PC(C), differed significantly to each other in their trueness (Friedman test: p < 0.001; Wilcoxon signed rank test: p < 0.001; Fig. 5). The CC(D) technique showed excellent agreement with the GS technique in all cases (median difference: 0.032, max: 0.262 mm^3^), suggesting this technique as appropriate for highly accurate tooth wear assessment.

**Figure 6:**
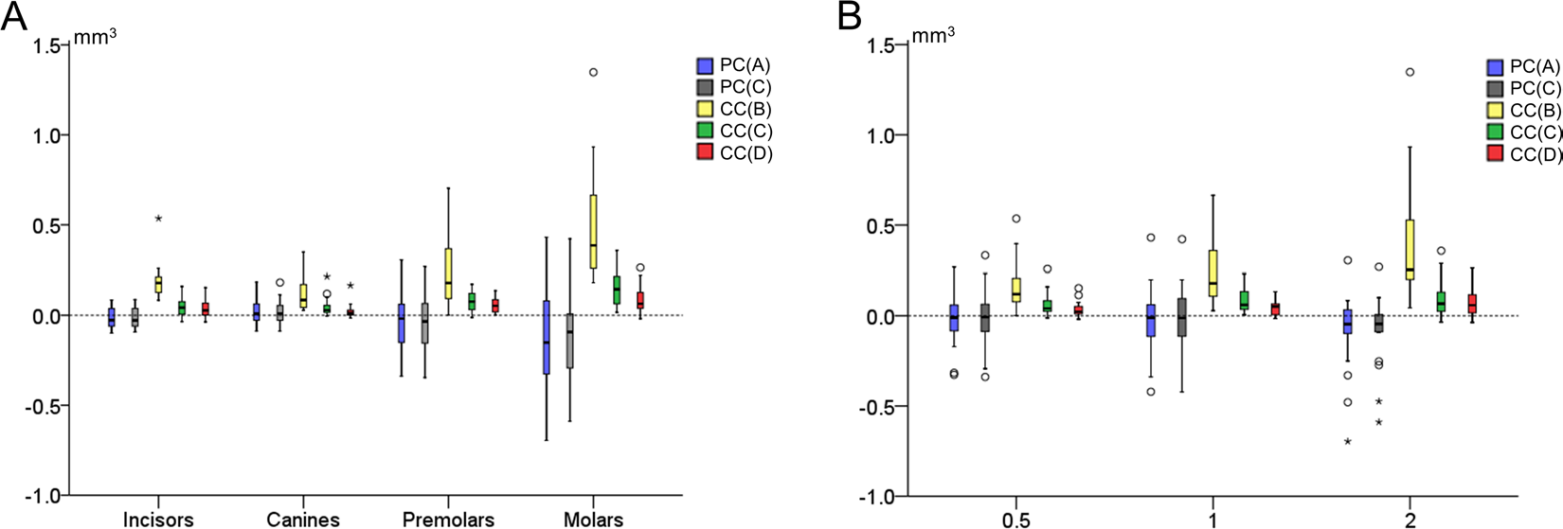
Box plots showing in the y-axis the difference of each technique from the gold standard technique in tooth wear measurements, (**A**) by tooth type and (**B**) by amount of tooth wear (vertical loss in mm). The upper limit of the black line represents the maximum value, the lower limit the minimum value, the box the interquartile range, and the horizontal black line the median value (trueness). Zero value indicates perfect agreement with the gold standard. The vertical length of each plot indicates precision.

Tooth type affected the trueness and precision of all techniques that use the complete crown as superimposition reference (CC; Kruskal-Wallis test, p < 0.01), with the premolars and molars showing the highest differences to the GS measurements (Fig. 6A). On the contrary, there was no difference between the trueness of each technique as measured in models with and without crowding (Mann-Whitney U test: p > 0.05). The amount of tooth wear affected significantly only the results of technique CC(B) (Kruskal-Wallis test, p = 0.001; Fig. 6B).

The difference of the technique of choice CC(D) from the GS technique in tooth wear measurements was always small for any tooth type or amount of tooth wear (Supplementary Figure S4). Significant differences were detectable only between canines and molars, with molars showing reduced trueness and precision (Mann-Whitney U test, p < 0.001). There was also a slight tendency for reduced trueness and precision when the amount of tooth wear was increased (Supplementary Figure S4, B).

## Discussion

This study presents an accurate and efficient method to assess tooth wear using hardware and software applications that are convenient to use and can be easily incorporated to the contemporary armamentarium of a regular clinical practice. The method is essentially a superimposition method, and the result can be visualized in all dimensions of space and quantified according to the clinician’s needs, as a linear or volumetric quantity. Apart from tooth wear, the present technique is expected to be suitable for dental material wear assessment, especially on the occlusal/incisal tooth surfaces, but this remains to be tested.

Previous studies questioned the laboratory defined performance of materials to that occurring clinically, among others also regarding tooth wear. This has primarily been attributed to the inadequacy of laboratory tests to simulate clinical conditions^19,20^. However, the lack of accurate clinical tooth wear assessment methods *in vivo* or *ex vivo*^11^ might have also confounded this relationship. The overall accuracy of the previously available wear assessment techniques has been reported to vary from 15 to 20 μm, regarding vertical loss, with high variation per tooth, per patient, and per study^11^. The presented technique showed an accuracy of 0.032 mm^3^, which is superior to previous techniques, considering that it refers to a volumetric measurement. The relation of volumetric to vertical loss is evident in Supplementary Figure S2. According to these data, the 0.032mm^3^ volume loss would correspond to approximately 9 μm of vertical loss.

According to a recent systematic review, most previous studies on tooth or dental material wear used special technical equipment and/or materials for the assessment of dental or material tooth wear *in vivo*, increasing the costs, the complexity, and the feasibility of the process, as well as the operator- or material-dependent effects on the outcomes^11,12^. Furthermore, these methods require the use of replicas and possibly indexes bonded to teeth, which add cost and error to the process^11^. The large amount of unusable data reported in previous studies is also indicative of the limitations of the currently available techniques^11^. Thus, apart from the high precision and trueness of the present technique, its major advantage concerns its applicability in everyday clinical conditions, since the required surface models can now be easily obtained by intraoral scanning. Numerous surface matching software applications have also become widely available, though their performance for such outcomes has to be individually tested.

The quality of the surface models inevitably affects the validity of tooth wear outcomes. Currently the intraoral scanners available on the market offer a global accuracy of approximately 30–50 μm^21,22^. However, errors are reduced when smaller structures, such as single teeth, which are required for the suggested technique, are considered^22,23^. The scanner used in the present study was a highly accurate (<20 μm) laboratory scanner in order to limit the effect of the scanner inaccuracy factor on the results. This accuracy is comparable or superior to that obtained from previously used techniques, such as profilometry^11^. The surface distance between corresponding points of repeatedly scanned dental models by this scanner is always less than 5 μm in all areas of the model. This accuracy level is slightly higher to the one that can be obtained from the currently available intraoral scanners, under actual clinical conditions. However, this design allowed for a gold standard measurement that provided the true value, which would have not been otherwise available^22^. In any case, with the very rapid advancement of intraoral scanners, it is expected that higher accuracy, which is preferable for valid 3D surface model superimposition outcomes^24^, will be achieved in the near future.

The matching software can be another important factor affecting tooth wear outcomes^11^. When registering identical 3D models, the software used in this study achieves accuracy higher than 0.1 μm. This software has been extensively tested previously for the processing and superimposing of surface models^22,24–26^ and proved to perform satisfactorily. The high repeatability of tooth wear measurements observed in the present study confirmed this finding.

A recent systematic review showed that most previous studies on tooth or dental material wear assessment reported 2D outcomes and mainly the vertical height loss^11^. It has been argued that 2D measurements might be more clinically relevant since they are not affected by tooth size^27^. We decided to report here volumetric measurements, since they provide higher amount of information. However, with the present technique and namely by the visualization of lost tooth structure through color coded distance maps, thousands of 2D measurements on the entire affected surface are available to the operator, both for visual assessment and quantification.

Moreover, although we tested here tooth wear assessment on the entire occlusal surface, the present technique is suitable for measurements at selective surfaces. These can be performed by isolating the volume of interest through slicing levels and hole filling processes that are similar to those reported here for the entire occlusal surface.

Regarding the reference areas used to register the serial 3D digital dental models, previous clinical studies followed various approaches, which primarily included the use of lingual or buccal surfaces or of occlusal surfaces that were considered free of occlusal contacts, based on the inspection of intraoral photographs^11,28^. Other researchers used software based approaches to detect stable superimposition reference areas, performing a quantitative or qualitative assessment of the distances between corresponding models, following an initial matching^5,11^. However, the validity of such approaches might be questioned, since proper reference area selection or serial 3D model matching cannot be guaranteed and it was not tested against a gold standard evaluation. Furthermore, most of the previous techniques included impression procedures, stone model construction, and special scanning equipment, which might, additionally, introduce bias to the outcomes. In the present study, after testing various potentially eligible reference areas and software settings towards a gold standard assessment it became evident that with certain settings, the complete tooth crown should be selected as a reference area to achieve results that are similar to the actual values. Thus, the operator-effect on the measured outcomes is expected to be minimized, since the reference area selection is straightforward and the matching process fully automated. Indeed, the operator effect was minimal as shown by the intra- and inter-operator reproducibility of the gold standard and the selected technique. The operator effect was evident in the present study, through the high variation of repeated measurements obtained using setting C and its lower trueness and precision compared to setting D. Although superimpositions with setting C were performed while viewing the overlap of intact structures, setting D, which was standardized, showed better performance.

### Limitations

The main limitation of the study was that the proposed technique was tested *in vitro* and not on actual patient data. However, the main problem with actual patient data is that a gold standard reference that provides the true condition, cannot be obtained. Thus, *in vitro* testing is so far the only way to test the validity of the technique. As discussed previously, we expect that the proposed technique will perform satisfactorily on actual patient data. Another limitation of the technique is that it may not be suitable for teeth that underwent morphological alterations over time, in parts of the crown other than the occlusal surface, which are used as superimposition references. Such circumstances could occur due to erosion, restorations, decay, or fixed retainer placement at the tested teeth. We suggest that during the superimposition process, the operator should assess this by testing the overlap of the superimposition reference areas though visualization of relevant color coded distance maps. In cases such as the one mentioned above, the registration of the subsequent models might not be optimal, and thus, the outcome might not be accurate. Future studies are needed to test the performance of the suggested technique in presence of such conditions.

## Conclusion

Utilizing 3D dental model surface data and standard 3D surface-based superimposition techniques we developed and tested here an efficient approach for *in vivo* occlusal tooth wear assessment, which can be easily implemented both for clinical and research purposes, using data obtained from high-performance intraoral scanners.

The study tested various superimposition reference areas and software settings and confirmed that these factors significantly affect the wear outcome.

According to our findings, the complete tooth crown should be selected as superimposition reference, using certain settings on a specific commercial software. The method is straightforward, easy to use and it shows increased accuracy when used on teeth that are not expected to undergo significant morphological alterations in parts other than the occlusal surface.

## Data availability

All outcome data are available as summary measures or representative images in the main text or the extended data. The surface models, protocols, and raw datasets generated and/or analyzed during the current study are available from the corresponding author on reasonable request.

## Supplementary information

Supplementary Information.

## References

[1] Lee A, He LH, Lyons K, Swain MV (2012). Tooth wear and wear investigations in dentistry. J. Oral Rehabil..

[2] Huysmans MC, Chew HP, Ellwood RP (2011). Clinical studies of dental erosion and erosive wear. Caries Res..

[3] Heintze SD, Cavalleri A, Forjanic M, Zellweger G, Rousson V (2008). Wear of ceramic and antagonist–a systematic evaluation of influencing factors *in vitro*. Dent. Mater..

[4] Stober T, Bermejo JL, Schwindling FS, Schmitter M (2016). Clinical assessment of enamel wear caused by monolithic zirconia crowns. J. Oral Rehabil..

[5] Eccles JD (1979). Dental erosion of nonindustrial origin. A clinical survey and classification. J. Prosthet. Dent..

[6] Smith BG, Knight JK (1984). An index for measuring the wear of teeth. Br. Dent. J..

[7] Hooper SM, Meredith N, Jagger DC (2004). The development of a new index for measurement of incisal/occlusal tooth wear. J. Oral Rehabil..

[8] Bartlett D, Ganss C, Lussi A (2008). Basic Erosive Wear Examination (BEWE): a new scoring system for scientific and clinical needs. Clin. Oral Investig..

[9] Al-Omiri MK, Sghaireen MG, Alzarea BK, Lynch E (2013). Quantification of incisal tooth wear in upper anterior teeth: conventional vs new method using toolmakers microscope and a three-dimensional measuring technique. J. Dent..

[10] Lee SP (2012). The development of quantitative methods using virtual models for the measurement of tooth wear. Clin. Anat..

[11] Wulfman C, Koenig V, Mainjot AK (2018). Wear measurement of dental tissues and materials in clinical studies: A systematic review. Dent. Mater..

[12] Heintze SD (2006). How to qualify and validate wear simulation devices and methods. Dent. Mater..

[13] Pintado MR, Anderson GC, DeLong R, Douglas WH (1997). Variation in tooth wear in young adults over a two-year period. J. Prosthet. Dent..

[14] Stucki S, Gkantidis N (2019). Assessment of techniques used for superimposition of maxillary and mandibular 3D surface models to evaluate tooth movement: a systematic review. Eur. J. Orthod..

[15] Panos PG, Tarawneh FM, Athanasiou AE (2011). Occlusal wear following orthodontic treatment assessed by 3D CT scanning. Orthodontics (Chic.).

[16] Park J (2014). A novel method for volumetric assessment of tooth wear using three-dimensional reverse-engineering technology A preliminary report. Angle Orthod..

[17] Tantbirojn D, Pintado MR, Versluis A, Dunn C, Delong R (2012). Quantitative analysis of tooth surface loss associated with gastroesophageal reflux disease. A longitudinal clinical study. J. Am. Dent. Assoc..

[18] Besl PJ, Mckay ND (1992). A Method for Registration of 3-D Shapes. IEEE Trans. Pattern Anal. Mach. Intell..

[19] Heintze SD, Faouzi M, Rousson V, Ozcan M (2012). Correlation of wear *in vivo* and six laboratory wear methods. Dent. Mater..

[20] Heintze SD (2017). Laboratory mechanical parameters of composite resins and their relation to fractures and wear in clinical trials-A systematic review. Dent. Mater..

[21] Vandeweghe S, Vervack V, Dierens M, De Bruyn H (2017). Accuracy of digital impressions of multiple dental implants: an *in vitro* study. Clin. Oral Implants Res..

[22] Winkler J, Gkantidis N (2020). Trueness and precision of intraoral scanners in the maxillary dental arch: an *in vivo* analysis. Sci. Rep..

[23] Ender A, Zimmermann M, Mehl A (2019). Accuracy of complete- and partial-arch impressions of actual intraoral scanning systems *in vitro*. Int. J. Comput. Dent..

[24] Henninger E, Vasilakos G, Halazonetis D, Gkantidis N (2019). The effect of regular dental cast artifacts on the 3D superimposition of serial digital maxillary dental models. Sci. Rep..

[25] Vasilakos G, Koniaris A, Wolf M, Halazonetis D, Gkantidis N (2018). Early anterior crossbite correction through posterior bite opening: a 3D superimposition prospective cohort study. Eur. J. Orthod..

[26] Vasilakos G, Schilling R, Halazonetis D, Gkantidis N (2017). Assessment of different techniques for 3D superimposition of serial digital maxillary dental casts on palatal structures. Sci. Rep..

[27] Stober T (2014). Comparability of clinical wear measurements by optical 3D laser scanning in two different centers. Dent. Mater..

[28] Heintze SD (2013). Wear of two denture teeth materials *in vivo*-2-year results. Dent. Mater..

