# Supplementary material for: An accurate and efficient method for occlusal tooth wear assessment using 3D digital dental models
Source: Sci Rep. 2020 Jun 22;10:10103. doi: 10.1038/s41598-020-66534-4 (PMC7308323; doi:10.1038/s41598-020-66534-4)
Supplement: Supplementary file 1 — Supplementary Information. [file 41598_2020_66534_MOESM1_ESM.pdf]

Supplementary material

An accurate and efficient method for occlusal tooth wear assessment using 3D digital dental models

Nikolaos Gkantidis, Konstantinos Dritsas, Yijin Ren, Demetrios Halazonetis, Christos Katsaros

Supplementary Table S1. Detailed report of the used dental models and teeth to simulate tooth wear.

Model No	Jaw	Aligned dental arches	Worn teeth<br>Tooth number (Wear amount in mm)				
1	Maxilla	No	15 (0.5)	13 (1)	11 (1)	24 (2)	26 (1)
2	Maxilla	No	16 (2)	13 (0.5)	11 (0.5)	22 (1)	26 (0.5)
3	Maxilla	No	15 (1)	13 (2)	22 (2)	24 (0.5)	
4	Maxilla	No	16 (1)	14 (2)	12 (1)	23 (1)	
5	Maxilla	Yes	16 (0.5)	12 (0.5)	21 (1)	23 (0.5)	25 (0.5)
6	Maxilla	Yes	14 (1)	11 (2)	22 (0.5)	24 (2)	
7	Maxilla	Yes	16 (2)	14 (0.5)	12 (2)	21 (0.5)	23 (2)
8	Maxilla	Yes	16 (1)	21 (2)	24 (1)	26 (2)	
9	Mandible	No	46 (0.5)	43 (1)	33 (0.5)	35 (2)	
10	Mandible	No	46 (2)	43 (0.5)	41 (2)	33 (1)	36 (1)
11	Mandible	No	46 (1)	44 (2)	42 (1)	33 (2)	36 (0.5)
12	Mandible	No	45 (1)	43 (2)	31 (0.5)	33 (1)	36 (2)
13	Mandible	Yes	45 (0.5)	43 (1)	34 (2)	36 (1)	
14	Mandible	Yes	46 (0.5)	42 (0.5)	33 (0.5)	35 (0.5)	
15	Mandible	Yes	46 (2)	43 (0.5)	41 (1)	33 (2)	35 (1)
16	Mandible	Yes	45 (1)	43 (2)	31 (2)	36 (0.5)	

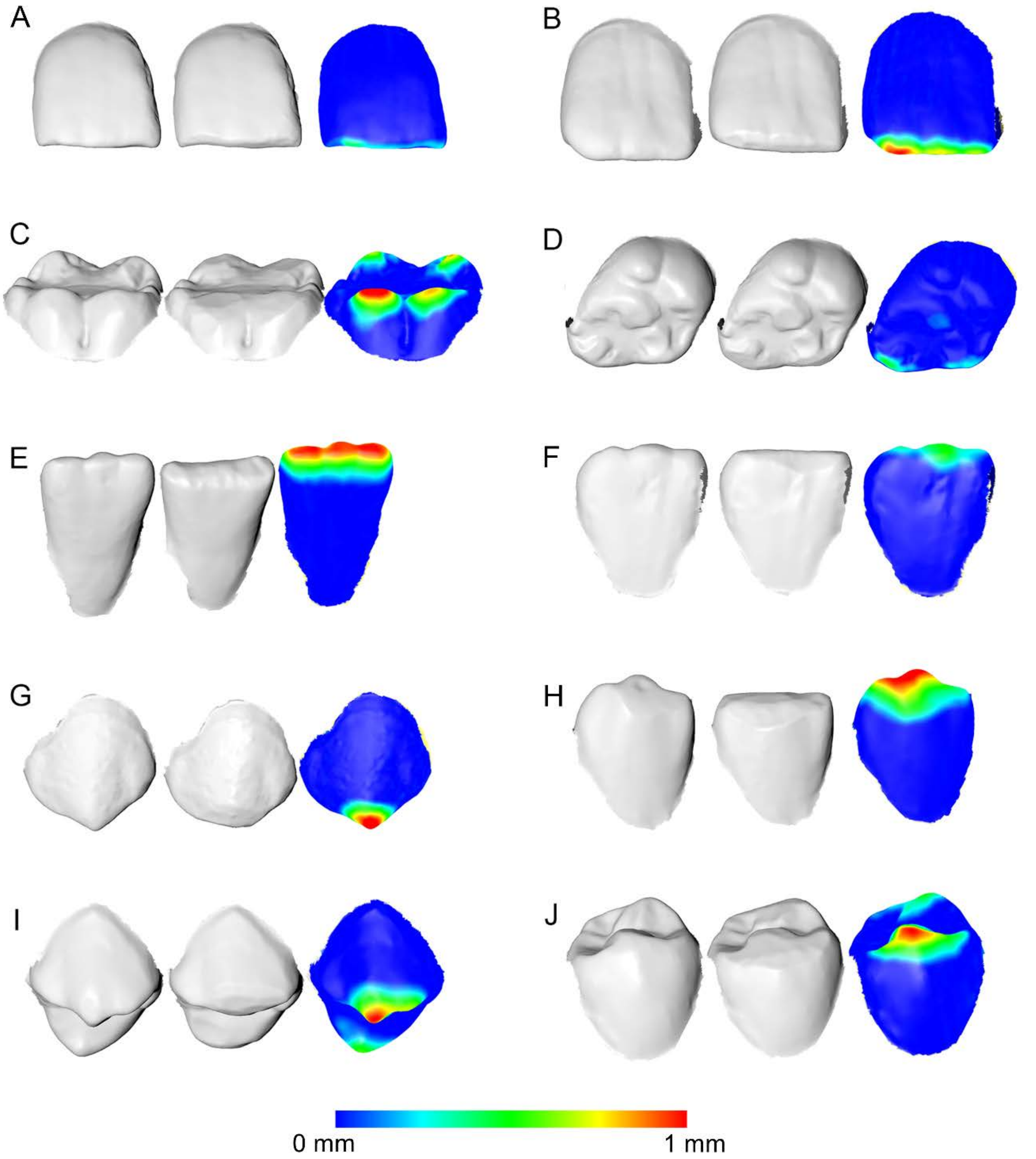

Supplementary Figure S1. Examples of teeth before (left) and after (middle) simulated tooth wear and colour coded distance maps (right) showing the missing tooth structure in each case. The colour maps were constructed following the superimposition of each before and after tooth wear condition, with the gold standard reference method used in the study. (A, B) Upper incisors. (C) Mandibular molar. (D) Maxillary molar. (E, F) Mandibular incisors. (G) Maxillary canine. (H) Mandibular canine. (I) Maxillary premolar. (J) Mandibular premolar. All images were generated using Viewbox 4 software (version 4.1.0.1 BETA, http://www.dhal.com/viewboxindex.htm).

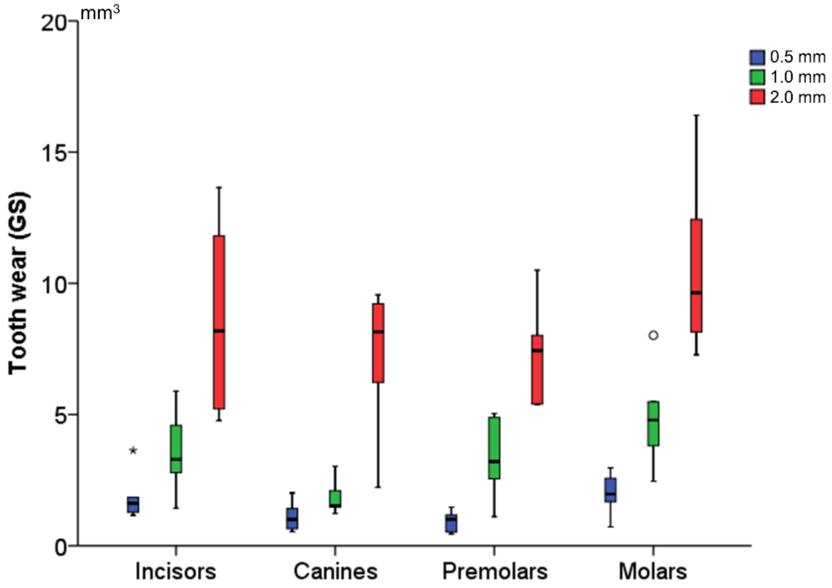

Supplementary Figure S2. Box plots showing in the y-axis tooth wear measurements by tooth type, measured with the gold standard technique. Colours show the planned vertical height reduction of the tooth cusps. The upper limit of the black line represents the maximum value, the lower limit the minimum value, the box the interquartile range, and the horizontal black line the median value. Note the consistent pattern of the measured tooth wear in each tooth type, according to the study setting.

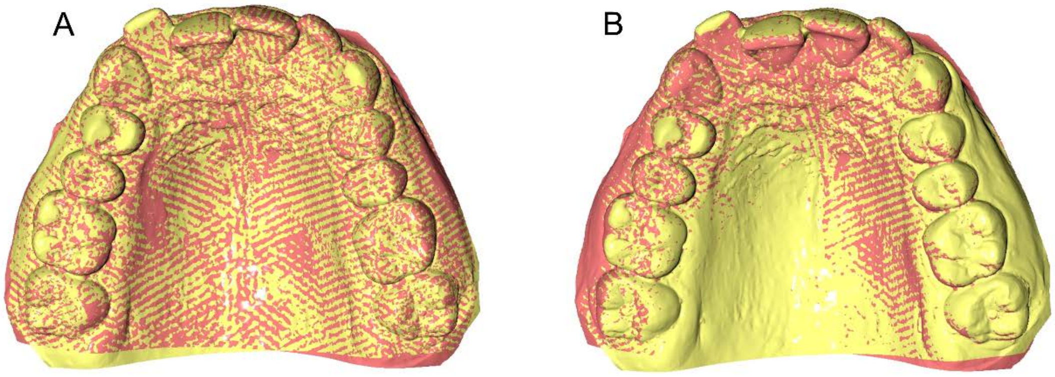

Supplementary Figure S3. Superimposed models before (red) and after (yellow) tooth wear using tooth 14 as superimposition reference. (A) Gold standard superimposition technique. Note the even distribution of the two colours in the whole surface suggesting the optimal registration of the two models in all areas. (B) Complete crown, setting D superimposition technique. Note that for tooth 14, which was used as reference, the overlap of the two models is satisfactory, but in other areas of the models the registration is inferior to that obtained with the gold standard technique. All images were generated using Viewbox 4 software (version 4.1.0.1 BETA, http://www.dhal.com/viewboxindex.htm).

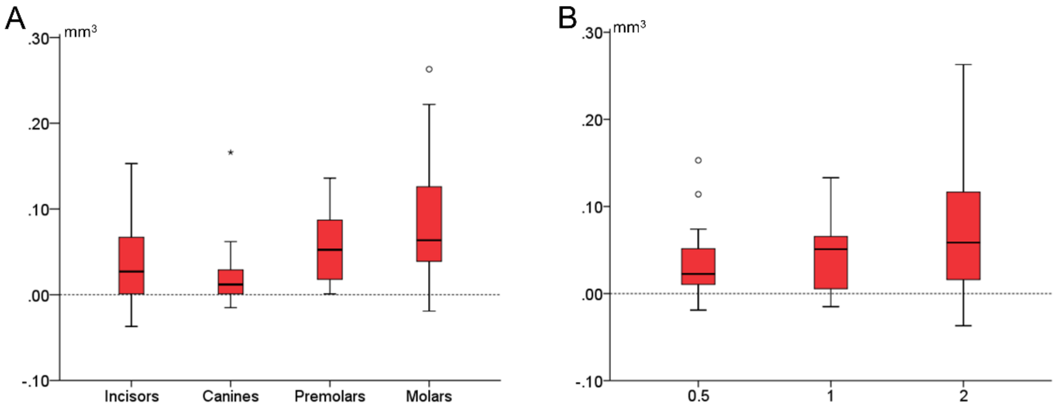

Supplementary Figure S4. Box plots showing in the y-axis the difference of the technique of choice (complete crown, setting D) from the gold standard technique in tooth wear measurements (A) by tooth type and (B) by amount of tooth wear. The upper limit of the black line represents the maximum value, the lower limit the minimum value, the box the interquartile range, and the horizontal black line the median value (trueness). Zero value indicates perfect agreement with the gold standard. The vertical length of each plot indicates precision.
